# An update on passive transport in and out of plant cells

**DOI:** 10.1093/plphys/kiab406

**Published:** 2021-09-04

**Authors:** Melissa Tomkins, Aoife Hughes, Richard J Morris

**Affiliations:** Computational and Systems Biology, John Innes Centre, Norwich Research Park, NR4 7UH Norwich, UK

## Abstract

Transport across membranes is critical for plant survival. Membranes are the interfaces at which plants interact with their environment. The transmission of energy and molecules into cells provides plants with the source material and power to grow, develop, defend, and move. An appreciation of the physical forces that drive transport processes is thus important for understanding the plant growth and development. We focus on the passive transport of molecules, describing the fundamental concepts and demonstrating how different levels of abstraction can lead to different interpretations of the driving forces. We summarize recent developments on quantitative frameworks for describing diffusive and bulk flow transport processes in and out of cells, with a more detailed focus on plasmodesmata, and outline open questions and challenges.

## Teaser

New insights enhance our understanding of the physical forces that drive passive transport across plant membranes highlights.

## Introduction

From the transport of energy and molecules across membranes, plants grow and develop, building complex 3D forms, responding to their environment and powering movement (Morris and Blyth, 2019; Ackermann and Stanislas, 2020; Cheung et al., 2020; Klejchova et al., 2021). These communication and transport interfaces are the key for everything that plants do and are critical for their survival. Transport and signaling across membranes are thus essential mechanisms and an appreciation of these processes is key for understanding how plants work.

**Advances**

The mechanisms underlying ion transport and signaling in plants are now well characterized. In particular, the fields of calcium signaling and guard cell ion dynamics have benefited from a long tradition of quantitative approaches coupled with mathematical modeling (for reviews, see Hedrich, 2012; Vaz Martins et al., 2013; Hedrich et al., 2016; Klejchova et al., 2021). We now understand such processes in great detail, the physical principles are well-defined and the modeling frameworks are advanced (Capoen et al., 2011; Hills et al., 2012; Evans et al., 2016; Liu et al., 2020). For instance, the multiscale extension of ion flux guard cell models with thermodynamics (Wang et al., 2017) demonstrates an impressive predictive power (Hills et al., 2012; Jezek and Blatt, 2017; Wang et al., 2017).

- The virial theorem provides a microscopic, energy-based explanation for osmotic flow and osmotic pressure.
- Molecular dynamics simulations of transport through aquaporins confirm the dominance of advection over diffusion for osmotic flow.
- Pressure is shown to reduce the SEL of plasmodesmata and differential pressure is demonstrated between neighboring cells.
- Pressure is shown to be different between cells and related to cell connectivity and topology.
- Advances in theoretical nanofluid dynamics coupled with computational approaches for modeling advection and diffusion in complex geometries show that channel flexibility can influence transport rates.

**Figure 1:**
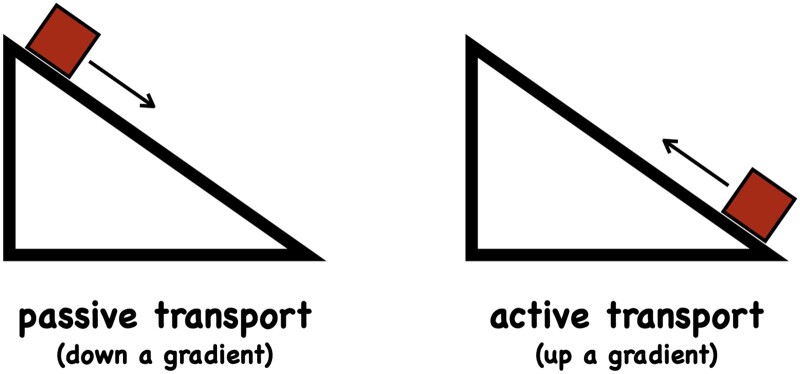
Transport requires energy. Unless prevented from doing so, systems out of equilibrium will tend to move back to equilibrium state, down a gradient (left), see also Boxes 1 and 2. Mechanically this equilibrium state corresponds to a net zero force and a minimum in the potential energy, statistically this equilibrium is a state of maximum entropy (subject to suitable constraints), see Box 2. Moving an entity out of a (mechanical or thermodynamic) equilibrium position against a counter-acting force, such as friction, viscous forces, gravity, electrostatic repulsion, etc., can be thought of as a climb up a gradient (right). Moving a particle up a gradient requires energy and is termed active transport. Heat transfer is the consequence of a temperature gradient (Fourier’s law), momentum is transferred when velocity gradients are present (Newton’s law), charge transport arises when there are electric potential gradients (Ohm’s law) and mass is transported if there are concentration gradients (Fick’s law) or pressure gradients (Navier–Stokes equation). Transport processes in which particles follow a gradient—typically a concentration (diffusion), pressure (mass flow, osmosis) or electric potential (electrical currents) gradient—are called passive. Cells invest large amounts of energy to build up and maintain such gradients in order for passive transport to occur. For instance, cells maintain huge free calcium gradients across the plasma membrane (a free calcium concentration ratio in the order of 10^4^–10^5^) with every calcium ion moved out of the cell requiring the hydrolysis of approximately one ATP molecule (around 56 kJ/mol for typical ATP/ADP ratios in the cell).

Here, we focus on advances in understanding the passive transport of molecules between cells (Figure 1). We give a brief overview of diffusion and then describe osmosis based on recent theoretical developments. While osmosis is an essential phenomenon for plant life and has been studied for well over 100 years, it is still frequently misunderstood (Reinke et al., 2020). As has been pointed out (Borg, 2003; Kramer and Myers, 2012; Kramer and Myers, 2013) and as a few Internet searches will reveal, osmosis is still often described incorrectly in both textbooks as well as in scientific publications. Common misconceptions are outlined in Kramer and Myers (2012) and include describing osmosis as diffusion (down a water concentration gradient) or osmosis being a special property of water. The origin of the confusion between osmosis with diffusion, possibly goes back to Fick’s famous paper on diffusion (Fick, 1855). Fick derived his laws of diffusion by direct analogy to Fourier’s equation of heat. His results have been shown to be a correct description of the expectation value for the concentration of a diffusing substance as a function of space and time, yet in deriving his equations Fick thought he was solving the problem of osmosis. Both directed diffusion and osmosis do rely on concentration differences but that does not imply the same mechanism. Another potential source of confusion may lie in the different frameworks used to describe osmosis. We first motivate the consideration of multiple physical modeling frameworks on the example of pressure. This example shows how different conclusions may be drawn from different descriptions of the same phenomenon. After contrasting different approaches for describing diffusion, we present recent insights into the microscopic cause of osmosis (the virial theorem; Borg, 2003; Bowler, 2017), we then focus on recent approaches for describing movement from cell to cell through plasmodesmata.

## Pressure as an example of seemingly conflicting inferences arising from different modeling frameworks

The inferred cause for a transport process is conditional on the chosen modeling approach and the parameters in the model. As an example of how different modeling frameworks can lead to different inferences, we mention here the origin of pressure.

Pressure is typically perceived as something mechanical, indeed its definition is force over area. The pressure of a gas can be understood as the exchange of momentum as particles hit the wall of a vessel and bounce off. The change in momentum mv (mass *m*, velocity *v*) per unit time leads to a force, *F* = d(*mv*)/d*t* and this force per unit area is defined as pressure, *p* = *F*/*A*. It is purely mechanical in origin. In a thermodynamic description, we can define the free energy of system, *F* = *U*–*TS*, where *U* is the internal energy, *T* is the absolute temperature, and *S* is the entropy. See Box 1 on entropy (Shannon, 1948; Denbigh, 1989; Lambert, 2002; Grandy, 2008; Balibrea, 2016). The partial derivative of the free energy *F* with *V* defines pressure, *p* = −∂*F*/∂*V*. The internal energy *U* of the system does not depend on volume *V*, and we are left with *p* = (*TS*)/∂*V* from which we conclude that pressure is purely entropic in origin. How can the same well-known physical entity be both purely mechanical and purely entropic? The resolution of this conundrum is well-known (see, for instance, Sokolov, 2010) and will not be reproduced here, the example serves merely to show how different frameworks lead to different and seemingly conflicting results for the exact same physical process.

BOX 1. Entropy is not disorder

There are various, related definitions of entropy: the phenomenological definition of Clausius (Δ*S*  =  Δ*Q*/*T*, which is defined only in thermodynamic equilibrium and where Δ*Q* is the exchanged heat and *T* the absolute temperature); the statistical definition of Boltzmann (in Planck’s notation *S* = *k*_B_ log *W*, where *k*_B_ is Boltzmann’s constant, log the natural logarithm, and *W* the “multiplicity”, i.e. the number of microstate configurations compatible with a macrostate); the ensemble phase space definition on Gibbs (*S* = −*k*_B_∑ *p*_i_ log *p*_i_, where *p*_i_ is probability of a microstate *i* and *k*_B_ is Boltzmann’s constant); the information theory definition of Shannon (*H* = −∑ *p*_i_ log *p*_i_, where *p*_i_ is the probability of event *i*); the variational inference approach of Jaynes (*S* = −*k*∑ *p*_i_ log *p*_i_, where *p*_i_ is the probability of a microstate *i* and *k* is the constant; Grandy, 2008). Different logarithms can be used which we do not distinguish here as they correspond only to a change in units. The above definitions are not unrelated and the deep connection between entropy, probability, and information is now well established (e.g. Maxwell’s demon). Entropy describes our lack of information (or uncertainty) about a system (Shannon, 1948) or, equivalently for a thermodynamic system, the dispersal of energy among microstates (Jaynes, 1957; Grandy, 2008). In none of these definitions, does entropy provide a measure of our perceived disorder of a system. Boltzmann’s entropy quantifies the number of ways a macroscopic state can be achieved (Grandy, 2008; Pressé et al., 2013; Balibrea, 2016). A change in entropy corresponds to a change in the multiplicities of microstates (probabilities) which is equivalent to the dispersal of energy among microstates (heat; Grandy, 2008). A system in thermodynamic equilibrium is defined by the macroscopic state that has the highest number of compatible microstates associated with it (the maximum entropy state, subject to given constraints—see Box 2; Grandy, 2008; Balibrea, 2016). There are cases where a maximum entropy configuration corresponds to our perceived sense of disorder (and low entropy configurations appear ordered) and there are cases where a maximum entropy configuration is perceived as a highly ordered state (and low entropy configurations appear disordered; Denbigh, 1989; Lambert, 2002). The classic examples of water–oil layer, supercooled water, or sphere packing are cases where the disorder analogy breaks down but maximum entropy arguments deliver the correct solutions (Jaynes, 1957).

## Diffusion is driven by thermal motion

**Figure 2:**
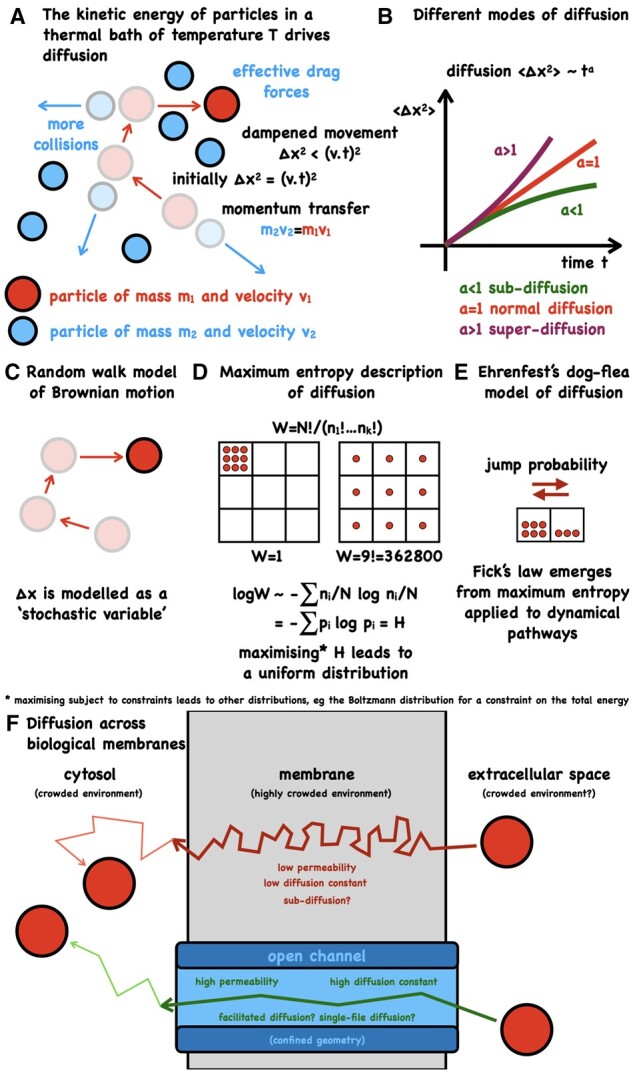
Diffusion is driven by thermal motion. (A) When particles collide they exchange momentum (*mv* with *m*=mass, *v*=velocity). The squared distance traveled by a particle moving with constant velocity is Δ*x*^2^ = (*vt*)^2^. Frequent collisions between particles results in a short mean free path length, viscosity and drag, leading to any movement being dampened. (B) Free diffusion of a solute in a solvent results in the well-known linear dependence of the expectation of squared traveled distance with time, <Δ*x*^2^> ∼ *t*, where the proportionality constant relates to the diffusion constant *D*. <Δ*x*^2^> = 2 *D t* holds for diffusion in one dimension (this factor of 2 becomes 4 for two dimensional and 6 for three dimensional domains). Crowded environments, such as the cytosol, lead to nonlinear behavior and subdiffusion. Superdiffusion is associated with an active transport process. (C) The erratic behavior of a single particle (Brownian motion) can be reproduced well by a random walk and Monte-Carlo simulation is a popular approach for finding particle trajectories. Mathematical equations for describing molecular diffusion include the Langevin equation (microscopic) and Fick’s law (macroscopic). (D) Entropy considerations lead to correct predictions for reproducible macroscopic states. Boltzmann’s entropy characterizes the number of ways a microstate can be realized. Shown here is the problem of distributing numbers of particles *n*_i_ in different locations *i*. There is only one configuration in which all particles are in one defined location, whereas a uniform distribution can be achieved in many more ways. (E) Ehrenfest’s famous dog-flea model of diffusion. Particles (fleas) all have the same jump probability from one side to the other (from dog to dog). The number of trajectories from the side with a high number of particles is greater than the reverse movement, providing a simple explanation for why a net flux of particles down a concentration gradient emerges from undirected thermal motion. Computing expectation values for the trajectories (maximum entropy on the trajectories = maximum caliber) leads to Fick’s equation, revealing the inference nature of the phenomenological transport equations. (F) Diffusion across a membrane is likely to be confined, crowded and anomalous, depending on the particles’ route.

Diffusion within plants provides an effective transport mechanism over short distances. Diffusion was first described by botanist Thomas Brown in 1827 based on his observations of the erratic movement of pollen in water (for a recent historical account of Brownian motion, see Genthon, 2020). Molecules in an environment with an absolute temperature above 0 have kinetic energy and are constantly in motion, colliding with other molecules in their vicinity (Figure 2A). As a consequence, molecules in a fluid are constantly being smashed into, following paths that without knowledge of these collisions may appear to be “random” (Brownian motion). When concentration (or thermal) gradients are present, the resulting higher number of trajectories leads to directed passive transport.

If the relationship  < Δ*x*^2^> ∼ *t* holds (the mean square displacement changes linearly with time), the process is termed normal diffusion (Sokolov and Klafter, 2005; Bressloff and Newby, 2013) and the proportionality factor (between  < Δ*x*^2^> and *t*) is related to the diffusion constant (Figure 2B). Einstein showed that the diffusion constant for a molecule in a fluid depends on the viscosity of the fluid, the temperature of the system, and the radius of the molecule (Einstein, 1905). We can thus assign the cause of diffusion to the kinetic energy of the solute molecules. We may also describe a diffusing particle as a random walk (Kac, 1947), abstracting from the underlying physics, and perhaps giving the impression that diffusion is driven by “randomness” of the diffusing particle or a probability distribution (Figure 2C). If we choose to use Fick’s law (Fick, 1855) for describing diffusion we find that concentration gradients or chemical potential differences are the source of diffusion. Maximum entropy arguments (Jaynes, 1957; Box 2) correctly predict the most probable state in which we would expect to find any given number of particles, subject to experimental constraints, and can also be used to analyze diffusion (Ghosh et al., 2006; Pressé et al., 2013; Figure 2, D and E). Using this framework might lead to the inference that diffusion is driven by the number of accessible configurations (entropy). While these interpretations can be reconciled (Grandy, 2008), it is well known that macroscopic descriptions do not determine the underlying microscopic laws and likewise that the microscopic laws by themselves do not lead to macroscopic states.

BOX 2. The principle of maximum entropy and transport phenomena

Although living systems operate far from thermodynamic equilibrium, this framework (and extensions thereof) can nevertheless represent a plausible approximation and be fruitful for developing insights. Thermodynamic equilibrium is the state of maximum entropy subject to any experimental constraints (macroscopic variables) by which we can control a system (Jaynes, 1957). If we change any of the macroscopic variables (change in information) and allow the system to reach a new thermodynamic equilibrium then that new state will be defined by maximum entropy under the constraints of those new conditions (Jaynes, 1957; Grandy, 2008). Building these ideas and the work of Gibbs and Shannon, Jaynes put forward the principle of maximum (information) entropy as a means assigning probabilities (Jaynes, 1957). Constraints can be added through the use of Lagrange multipliers. For instance, maximizing entropy subject to the condition that all probabilities must sum to one, leads to a uniform distribution, maximizing entropy subject to the additional constraint of knowing the total energy of the system leads to the Boltzmann distribution, including also a constraint on particle numbers leads to the grand-canonical distribution, as well as the standard terms for free energy and the chemical potential (Jaynes, 1957). Entropy and probabilistic inference are extremely powerful approaches, yet stating that something is driven by entropy is perhaps a little misleading and provides no insight into what is happening mechanistically or the real driving force. The maximum entropy principle is not limited to states and can be applied also to trajectories (often termed “maximum caliber” for dynamical systems analysis). Maximum entropy/caliber arguments have been used to derive the equation of Brownian motion, Fick’s law of diffusion, Fourier’s heat equation, the Navier–Stokes equations, as well as two-state systems and enzyme kinetics (Ghosh et al., 2006, 2020; Grandy, 2008; Stock et al., 2008; Pressé et al., 2013; Dixit et al., 2018).

In crowded environments such as the cytosol or membranes, the linear relationship between mean square displacement and time can break down, leading to anomalous diffusion (Sokolov and Klafter, 2005; Figure 2, B and F). Typically, this leads to a dependence  < Δ*x*^2^> ∼ *t*^a^ where *a* < 1, called subdiffusion. This phenomenon has arisen also in diffusion through narrow domains (channels and pumps) in which the diffusing particle interacts with the boundaries or where passage of diffusing particles past one another is hindered (Pagliara et al., 2014). There are several computational approaches for modeling anomalous diffusion that include continuous-time random walks and generalized Langevin equations (Vitali et al., 2018). The Langevin equation describes the position of a particle over time using a stochastic differential equation (Bressloff and Newby, 2013). Generalized Langevin methods have been employed for computing diffusion in complex media (Vitali et al., 2018) and transmembrane diffusivity (Gaalswyk et al., 2016). A recent development with a generalized Brownian but non-Gaussian model is presented in Sposini et al. (2018). The transition to superdiffusion, *a* > 1, has been studied using a Lévy process (Miron, 2020) and is associated with active transport (Reverey et al., 2015; Chechkin et al., 2017). Detailed descriptions of the physical ideas with the associated mathematics of stochastic models can be found in the excellent review by (Bressloff and Newby, 2013). Continued advances in spatiotemporal live imaging, chemical sensors, fluorescence markers, and statistical data analysis will be the key for quantifying various transport processes, characterizing their nature, and developing mathematical models.

## Osmotic flow is driven by pressure differences on the solvent molecules on either side of a semipermeable membrane

Osmosis is the key for plant life and contributes to maintaining cell form and function, cell growth, plant movement, and various transport processes. Osmosis was first described in the 18th century, by Jean-Antoine Nollet, although it was Henri Dutrochet in the 1820s who provided one of the first clear demonstrations of the phenomenon in plants (for a more complete history with recent developments, see Marbach and Bocquet, 2019).

**Figure 3:**
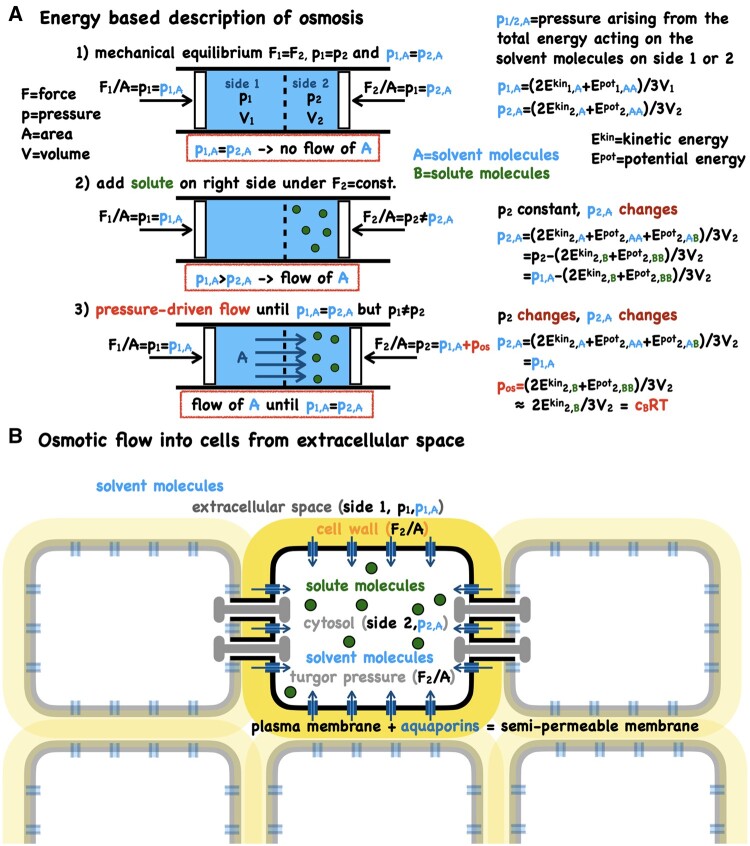
The virial theorem provides a mechanistic explanation for osmosis. (A) The virial theorem allows for pressure to be expressed via the kinetic, *E*^kin^, and potential energy, *E*^pot^, in the system (Borg, 2003; Bowler, 2017). The forces on sides 1 and 2 that are required to balance the pressure are denoted by *F*_1_ and *F*_2_, and the pressures acting on the solvent on either side by *p*_1A_ and *p*_2A_ (these are not partial pressures). The individual steps 1, 2, 3 and the corresponding energetic changes are given in the figure. Note that the pressure differential on the solvent depends on the interaction term between solvent and solute but that the resulting total pressure difference (the osmotic pressure) depends only on the kinetic energy of the solute and the (usually negligible) solute-solute interaction energy. Neglecting solute-solute interactions results in the van’t Hoff approximation, *p*_os_=*c*_B_*RT*, in which *c*_B_ is the molar concentration of the solute, *R* the universal gas constant and T the absolute temperature. These energetic considerations make clear that osmosis is not a special property of water and indeed osmosis occurs also in gases. Whilst molecular diffusion will be present it is not a significant contributor. A net diffusive flux would require a solvent concentration gradient which is often not present (and can even go the other way depending on solvent–solute interactions). Thus, diffusion or facilitated diffusion are not the drivers of osmotic flow. (B) In biological systems, pressure-driven water transport occurs through water channels (aquaporins). In plants, plasmodesmata may also act as water channels between cells (Figure 4). Aquaporins and plasmodesmata can be regulated (gated) in a variety of different ways, demonstrating the genetic control of the plant’s implementation of the semipermeable membrane (the nature of the semipermeable membrane is ignored in the thermodynamic idealization and also in the above derivation of osmosis).

Thermodynamically, osmosis flow can be viewed as an entropic effect. The mixing of two substances (solvent and solute) increases the entropy which results in a difference in the chemical potential between the two compartments on either side of a semipermeable membrane (Borg, 2003; Marbach and Bocquet, 2019). Differences in chemical potential drive the flux of particles, until the chemical potentials are equal. The entropy of mixing via the chemical potential thus appears as the thermodynamic driving force for this process but it says nothing about the actual mechanism. This has led to some imaginative microscopic interpretations and much controversy (Borg, 2003; Alleva et al., 2012; Kramer and Myers, 2012). We need to go to microscopic descriptions of the system to get to the mechanism and physical driving force (Figure 3). Osmotic flow is driven by a pressure difference acting on the solvent between the two sides of a semipermeable membrane and this pressure difference arises from changes in the kinetic and potential energies caused by the addition of the solute (Bowler, 2017; Figure 3A). This can also be understood by considering that the semipermeable membrane has a repulsive potential energy for the solute but not the solvent (Kramer and Myers, 2013; Bowler, 2017; Marbach and Bocquet, 2019).

The power of thermodynamics is that it makes macroscopic predictions without the need for these microscopic details—this can also be a limitation.

From the general equation for osmotic pressure (Bowler, 2017), the well-known van’t Hoff equation emerges for the case when the potential energy interaction terms between solute molecules is 0 (i.e. far apart). Noninteracting particles obey the requirements of an “ideal gas” for which the pressure is given by *p* = *cRT*, where *c* is the concentration of the particles, *R* is the universal gas constant, and *T* is the absolute temperature (Marbach and Bocquet, 2019).

Although the nature of the semipermeable membrane does not enter into any of the above considerations or the standard theoretical frameworks for describing osmosis, it is clearly important as without the semipermeable membrane there would be no osmosis flux nor osmotic pressure (Figure 3B). The membrane permeabilities for solutes and the solvent are the key parameters. Aquaporins have been shown to be major routes for water transport across membranes, with fluxes exceeding those expected for diffusion across a lipid bilayer by orders of magnitude. Aquaporins can transport 10^9^ water molecules per second (Jensen and Mouritsen, 2006). Recent atomic simulations of aquaporin transport have reconfirmed that pressure flow is the dominant form of transport (Horner and Pohl, 2018; Kepner, 2018), characterized by the ratio of osmotic permeability over diffusive permeability (a quantity akin to the Péclet number; van de Meent et al., 2008; Box 3). Estimates of this ratio vary but are typically greater than 10 (Jensen and Mouritsen, 2006; Wambo et al., 2017). Aquaporins are gated via phosphorylation as demonstrated by detailed molecular dynamics simulation on structures determined by x-ray crystallography (Törnroth-Horsefield et al., 2006; Alleva et al., 2012; Kapilan et al., 2018; Fortuna et al., 2019; Singh et al., 2020). Several other regulatory mechanisms of aquaporins in plants have been described (Alleva et al., 2012; Fortuna et al., 2019; Kapilan et al., 2018; Singh et al., 2020); thus, adding further layers of complexity compared with the thermodynamic idealization.

BOX 3. The Péclet number quantifies bulk flow over diffusion

Molecular diffusion can be expected to occur in any fluid. The < Δ*x*^2^> ∼ *Dt* dependency of diffusion (i.e. the expected time to travel a given distance, Δ*x*, varies with the square of distance) makes this mode of transport efficient over short distances (relative to *D*^1/2^) but increasingly inefficient for larger distances. For instance, for a molecule with a diffusion constant of *D* = 10^−10^ m^2^ s^−1^ (100 μm^2^ s^−1^) to move a distance of Δ*x* = 10 μm would take approximately *t* = 0.5 s and a distance to 1 cm would take approximately 6 d. Advection (mass flow) is an effective way of enhancing transport. A useful way of characterizing flow based on these two modes of transport is the Péclet number. The Péclet number is defined as a dimensionless ratio of the rates of advection over diffusion (van de Meent et al., 2008). For mass transfer, the Péclet number is related to the Reynolds number (Purcell, 1977). The Reynolds numbers characterizes fluid flow by the ratio of inertia and viscous forces (Jensen et al., 2016). For fluid flow in plants, the Reynolds number will typically be significantly less than 1 (Re < 0.001 for phloem flow, Re < 0.1 for xylem flow), meaning that the flow is laminar and dominated by viscous forces (Jensen et al., 2016). The Péclet number is important for characterizing passive transport processes and some unexpected effects depend on this number. For instance, Taylor dispersion, or shear-enhanced diffusion, describes the effects of flow on diffusion which can lead to a significant increase in the effective diffusion, as shown for xylem flow (Blyth and Morris, 2019).

From the above considerations we should expect that whenever there is a difference in concentrations of molecules that are too large to move through channels, pores, or plasmodesmata (thus, making the interface between compartments act like a semipermeable membrane), there should be a difference in pressure between compartments/cells and an associated flow until an equilibrium is reached for those molecules that can move freely through the membrane (typically the solvent).

## Osmosis and diffusion are important for cell-to-cell transport

A key route for transport and signaling between plant cells is through plasmodesmata (Faulkner, 2018; Li et al., 2021). Plasmodesmata were first described by Eduard Tangl in 1879. For a recent historical overview on plasmodesmata research see van Bel (2018). A broad range of cellular processes depend on this symplastic transport, yet the mechanistic details of the transport process remain elusive (Hernández-Hernández et al., 2020; Petit et al., 2020; Li et al., 2021). Plasmodesmata can have different geometries that can be influenced, potentially fine-tuned, via callose deposition/degradation (for instance, during infection; Cheval and Faulkner, 2018) and likely permit different modes of transport.

**Figure 4:**
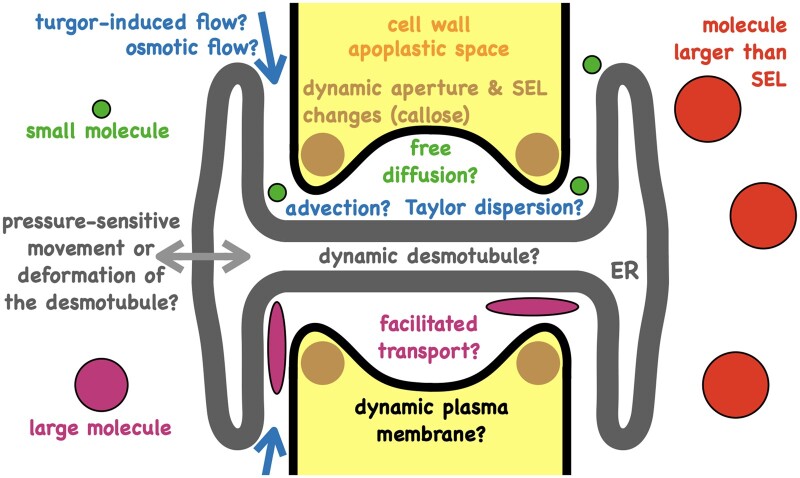
Plasmodesmata may allow for different modes of transport between cells. Small molecules are likely to be able to diffuse through plasmodesmata. This is usually modeled as normal diffusion with or without geometrical hindrance factors. If pressure differences exist between cells then advection may occur. For larger molecules the mode of transport remains unclear. Selective molecular transport through plasmodesmata may lead to concentration differences (of those molecules that cannot pass) between neighboring cells and potentially to osmotic flows through plasmodesmata, giving rise to osmotic and turgor pressure differences. Turgor pressure differences may lead to plasmodesmata closure via desmotubule/membrane dynamics. Depending on the transport route, turgor pressure or osmotic pressure may be more important which may give rise to some interesting flows and feedbacks. The precise modes of transport and their dependence on pressure, flow, dynamics of the plasma membrane, dynamics of the desmotubule, the associated proteins, and interactions between plasma membrane and the desmotubule remain to be characterized. SEL = size-exclusion limit (the maximum size of a molecule up to which movement through plasmodesmata is possible); ER = endoplasmic reticulum.

Shape, size, and density of plasmodesmata vary greatly between tissue and cell types (Nicolas et al., 2018). Modeling has mostly focused on simple, nonbranched plasmodesmata (Peters et al., 2021). In these types of plasmodesmata, the structure consists of a cytoplasmic sleeve between the plasma membrane that lines the pore and the membrane of the desmotubule (part of the endoplasmic reticulum that bridges the cytosol of neighboring cells; Figure 4). The geometry of the cytoplasmic sleeve is such that small molecules can likely diffuse through it. Various models for diffusion of molecules through the cytoplasmic sleeve have been developed. Recent work by Deinum et al. (2019) builds a detailed geometric model of plasmodesmata and uses various hindrance factors to account for the restricted environment of the diffusing molecules. Restrictions to normal diffusion include steric hindrances and interactions with other molecules (for instance, membranes or large protein complexes such as tethers that are present in the cytoplasmic sleeve). Diffusive hindrance could provide part of the explanation for the drastic reduction in diffusivity observed in similar models where plasmodesmata transport is modeled as diffusion, but requires a modified rate of diffusion for models to recapitulate experimental data. Alternative approaches that lead to modified or effective diffusion, resulting from Brownian particles in a confined volume with small exit areas (Holcman and Schuss, 2014; Grebenkov and Oshanin, 2017), have been suggested that model plasmodesmata as nonreflecting boundaries (an escape pore; Calderwood et al., 2016; Hughes et al., 2020, 2021).

The importance of plasmodesmal fluxes has recently been demonstrated for auxin flows (Gao et al., 2020; Mellor et al., 2020), that is, even for the case where specialized active transporters are present. Mellor et al. (2020) used a set of ordinary differential equations to describe the auxin concentration in root cells. Through a cycle of model predictions and experimental validation, they found that without accounting for flux through plasmodesmata observed concentration profiles could not be reproduced. Passive diffusion of auxin through plasmodesmata was found to be an important component of establishing auxin gradients within the root with plasmodesmata density emerging as a key parameter (Mellor et al., 2020). Gao et al. (2020) demonstrated that plasmodesmata can be regulated asymmetrically on a cellular level, leading to preferential intercellular transport along the midrib zone in the abaxial epidermis of Arabidopsis (*Arabidopsis thaliana*) leaves. Describing transport as a diffusion process, they computed local effective diffusion tensors in different areas of the leaf. Importantly, they show that not only is diffusion asymmetric but that within the same cell, different directions can have different permeabilities. This differential control of transport may be important for changing the local concentrations of defense compounds (Gao et al., 2020). The interplay between passive transport and active transport remains to be elucidated, also whether different concentrations of molecules that cannot pass through plasmodesmata may induce osmotic flows between cells.

The space between the cytoplasmic sleeve and the desmotubule of plasmodesmata can prevent some molecules from passing into the channel (Faulkner, 2018). Molecules that are larger than the so-called size-exclusion limit (SEL; typically measured in molecular weight) cannot traverse plasmodesmata passively. If some molecules (water, ions, small metabolites, small macromolecules) can pass but others not (large metabolites, macromolecules), then we have an instantiation of a semipermeable membrane. This may potentially mean that any changes to the population of molecules above the SEL in connected cells might induce an osmotic flow between the cells. Furthermore, recent observations suggest that neighboring cells can be under different turgor pressure and this was recapitulated in a model that showed this pressure differential to depend on their tissue connectivity (Long et al., 2020).

With different turgor pressures and potentially different osmotic pressures between neighboring cells, we can expect pressure-driven flows (advection) to occur (Anisimov and Egorov, 2002), yet increases in turgor pressure are known to reduce cell-to-cell movement (Oparka and Prior, 1992). This pressure-induced reduction in permeability is rapid and difficult to reconcile with a transport model of static geometry and the time-scales of callose deposition, suggesting a role for plasmodesmal dynamics that operate on faster timescales. Based on this premise, Park et al. (2019) developed an elastic spring model of the desmotubule, which via pressure-induced movement can change the area available for flow and/or diffusion through plasmodesmata. They assumed a small Péclet number (the rate of advection over diffusion, Box 3) and then, focusing on particle diffusion only, showed that the hindrance factor to transport increases slowly with pressure for pressure differences Δ*p* < 150 kPa and then rapidly for pressure differences Δ*p* > 150 kPa with complete blockage at Δ*p* = 225 kPa due to steric hindrance prior to closure. Using the van’t Hoff equation, it can be shown that Δ*p* = 150 kPa corresponds to a change in solute concentration of approximately 0.06 M (for *T* = 300 K). Both these pressure and concentration values depend on the geometrical and mechanical parameters in the model.

A model based on the underlying physics of advection and diffusion with realistic geometries was recently used to investigate phloem loading of sugars through plasmodesmata (Comtet et al., 2017). Shear-enhanced diffusion has been suggested to be important for xylem transport (Blyth and Morris, 2019; Box 3). Similar mechanisms may be active in aquaporins and plasmodesmata. Of interest to this problem is a recent development that uses transfer functions to build a general analytical model for advection coupled with diffusion in cylindrical environments (Schäfer et al., 2021). The extent to which advection and diffusion interact could potentially depend on the mechanical properties of the conduit and membrane dynamics. Recent work on artificial tubes has uncovered mechanisms that may be relevant for flows in biological systems. Marbach et al. (2018, 2020) investigated how fluctuations in the membrane structure affect flow. They demonstrate that “surface wiggling” enhances diffusion via induced hydrodynamic flows (Taylor dispersion) but that variation in the pore geometry can introduce trapping effects that can slow down the flow (often called entropic barriers). These modulations to the membrane surface could be thermal or induced by other mechanisms. They show that for Péclet numbers > 1 (Box 3), fluctuation in membrane (both thermal and induced) is expected to enhance transport. For Péclet numbers < 1, their models predict a decrease in transport. For aquaporins, Péclet numbers can vary between 10^−2^ and 10^2^ and indeed both predicted behaviors have been observed. Transport could in principle be tuned by adjusting the geometrical and dynamical properties of the channel-defining structures. How important such processes may be for plasmodesmata remains to be investigated and will depend on the interplay between osmosis, pressure flows, diffusion, membrane, and desmotubule dynamics.

Intercellular diffusion is likely to occur for small molecules and depending on the presence of concentration gradients this may or may not result in net fluxes. Advection requires a pressure difference (or a temperature difference) which could be caused by different turgor pressures between cells and osmosis. Some molecules above the SEL (typically proteins, transcription factors, or messenger RNA) are also transported between cells through plasmodesmata (Guenoune-Gelbart et al., 2008; Ueki et al., 2010; Paultre et al., 2016; Li et al., 2021). It is not clear how this happens and is typically put under the umbrella of changing the SEL, which could mean altering the geometry or material properties of the neck region and/or the cytoplasmic sleeve and/or the desmotubule and/or producing secondary plasmodesmata and/or the shape or properties of the molecule that is being transported.

Varying reports on the importance of diffusion over advection for plasmodesmal transport exist, suggesting this may be different depending on the species, tissue type, developmental stage, and type of plasmodesmata (Comtet et al., 2017; Ross-Elliott et al., 2017; Park et al., 2019; Hernández-Hernández et al., 2020). Furthermore, modification of the aperture by callose deposition changes the SEL, implying that molecules which could previously move from cell to cell are no longer able to do so (Faulkner, 2018). This in turn potentially increases the number of chemical species that could be considered to be osmolytes; thus, changing the osmotic pressure but also modifying the geometry that affects advection and diffusion. For instance, if the sugar concentration was higher in one cell relative to a neighboring cell and if sugars were not blocked from passing through plasmodesmata, then the cell with higher sugar concentration would lead to an osmotic flow into that cell from the extracellular space (as sugars would act as an osmolyte between inside and outside that cell) but not to influx from the neighboring cell (as plasmodesmata with a large enough SEL would not act as a semipermeable membrane for sugar). However, the resulting increase in turgor pressure in the cell with sugars might then lead to mass flow to its neighboring cell or pressure-induced plasmodesmata closure (Park et al., 2019), which might lead to an osmotic influx from the neighboring cell until an increased pressure reopens the plasmodesmata. The uncertainties and potential confusion of this paragraph demonstrate that there is still much that we (the authors) do not understand about the regulation and mode of plasmodesmal transport (Figure 4). Related points are discussed in excellent recent reviews (Nicolas et al., 2018; Petit et al., 2020; Li et al., 2021; Peters et al., 2021). Advanced computational models with smooth (i.e. no abrupt changes that may lead to numerical instabilities or cause potential artifacts such as the onset of turbulence), complex (taking into account key geometrical features), and potentially dynamic (capturing pressure-induced changes and fluid–structure interactions) geometries may help explore different transport scenarios and make predictions that can be validated/falsified with new experiments.

## Summary and outlook

Membrane transport keeps cells alive. Transport and signaling across membranes are of fundamental importance for responding to environmental stresses and coordinating plant growth and development. Understanding how plants respond to changes in their environment therefore requires a detailed understanding of the processes governing transport within and between cells. Yet, much remains to be uncovered with respect to these processes, their underlying physics, their regulation, and the relative contributions of different pathways or modes of transport across membranes or from cell to cell (e.g. active versus passive; aquaporin or transporter-mediated versus plasmodesmal; advection versus diffusion).

Here, we have shown how our perception of the mechanisms behind transport processes depends on the level of abstraction at which we view them, and we discussed how this can limit comprehension, or even produce erroneous descriptions.

If we view diffusion and osmosis through a thermodynamic lens, we would see that both processes are driven by entropy (or concentration differences or differences in chemical potential), and might therefore consider them to be the same. However, the actual mechanisms are quite different: in the first case the solute molecules diffuse due to thermal motion and statistically will tend to go down a solute gradient (as there are more possible trajectories from a high to low particle numbers), in the other case we have solvent molecules flowing, driven by a pressure gradient, up a solute gradient; one process is thermodynamically irreversible whilst the other is reversible (reverse osmosis).

Membrane transport and its impact on plant growth and development is a multiscale problem (Wang et al., 2017; Couvreur et al., 2018; Hubbard et al., 2020). Microscopic models can be used to shed light on detailed dynamic behavior (Jensen et al., 2016; Vaz Martins et al., 2016; Jezek and Blatt, 2017). Thermodynamics can be used effectively to constrain kinetic models so that they do not violate physical principles (Pan et al., 2019). Approaches such as maximum entropy procedures and maximum caliber are versatile and effective ways of bridging scales and integrating macroscopic observables and constraints with microscopic detail (Stock et al., 2008; Pressé et al., 2013; Dixit et al., 2018; Ghosh et al., 2020). Challenges to multiscale models of biological tissues have been outlined (Enkavi et al., 2019; Fletcher and Osborne, 2021). See “Outstanding questions”. Perhaps with advances in imaging coupled with expanding our repertoire of computational and mathematical tools that cover a range of different spatial and temporal scales (from microscopic to macroscopic, from mechanistic to statistical, from cell to tissue), we can improve our understanding of transport processes in plants further and reach new levels of predictive power.

**Outstanding questions**

- How can computational methods bridge spatial and temporal scales to develop a more comprehensive understanding of membrane transport processes?
- How might membrane dynamics (and fluid–structure interaction) contribute to plasmodesmal transport and how can we investigate this in a computationally tractable way?
- How can links between statistical physics, information theory, and inference (and perhaps machine learning) be exploited to integrate between different scales and different levels of approximation of transport processes?
- What technologies would we need to visualize and quantify permeabilities and fluxes in vivo?

## Notes

R.J.M. and M.T. conceived the idea for the manuscript. R.J.M., M.T., and A.H. jointly reviewed the literature and discussed key concepts. R.J.M. prepared the figures and wrote the manuscript with contributions from M.T. and A.H.

The author responsible for distribution of materials integral to the findings presented in this article in accordance with the policy described in the Instructions for Authors (https://academic.oup.com/plphys/pages/general-instructions) is Richard J. Morris (richard.morris@jic.ac.uk).
